# Comparison between dual-energy X-ray absorptiometry and skinfold thickness in assessing body fat in overweigh/obese adult patients with type-2 diabetes

**DOI:** 10.1038/s41598-017-17788-y

**Published:** 2017-12-12

**Authors:** Elisabetta Bacchi, Valentina Cavedon, Carlo Zancanaro, Paolo Moghetti, Chiara Milanese

**Affiliations:** 1 Section of Endocrinology, Diabetes and Metabolism, Department of Medicine, University and Azienda Ospedaliera Universitaria Integrata of Verona, Verona, Italy; 2 Department of Neurosciences, Biomedicine and Movement Sciences, University of Verona, Verona, Italy

## Abstract

Percentage of body fat (%BF) is estimated in clinical practice using anthropometric equations, but little is known about their reliability in overweight/obese patients with type-2 diabetes. The aim of this study was to compare, in overweight/obese adults with type-2 diabetes, %BF estimated with several commonly used anthropometric equations and %BF measured with dual-energy X-ray absorptiometry (DXA, Hologic). The %BF was measured with DXA in 40 patients aged 40–68 years with type-2 diabetes (mean HbA1c, 7.3 ± 0.9%). Body density was estimated in the same patients by means of four anthropometric equations and converted to %BF using the Siri and Brozek equations. Paired-sample t-test and the mean signed difference procedure were used to compare anthropometric equation-derived %BF and DXA measurements. The coefficient of determination was computed. Bland-Altman analysis was used to test the agreement between methods. Among the four anthropometric equations, the Durnin-Womersley equation only showed close agreement with DXA in both female and male patients; the other equations significantly underestimated or overestimated %BF. Two new predictive equations were developed using DXA as the reference to predict total body and trunk %BF. Further comparative studies are required to confirm and refine the accuracy of practical, non-invasive methods for monitoring %BF in this population.

## Introduction

Type-2 diabetes is a very common metabolic disorder characterized by hyperglycemia, which is related to insulin resistance and relative insulin deficiency^1^. Excess body fat (BF) contributes to impairing insulin action and is thought to be the primary factor underlying the global epidemic of type-2 diabetes which occurs worldwide. Consistently, rates of type-2 diabetes have increased markedly in parallel with those of obesity^2^. Moreover, excess BF is among the strongest risk factors for cardiovascular morbidity and mortality in type-2 diabetes patients^3–5^. Therefore, in clinical practice, an accurate assessment of body composition is a crucial point in the management of diabetic patients, and should be used for monitoring the effects of lifestyle interventions in these subjects.

BF can be assessed using different methods and tools, with variable costs, practicality and accuracy. Dual-energy X-ray absorptiometry (DXA) is considered to have high accuracy in estimating fat mass and fat-free mass^6,7^ but is not easily always available in the clinical setting. A common alternative is skinfold thicknesses, which is a simple and inexpensive approach to objectively assess BF^8–10^. In this regard, a number of different regression equations have been developed to estimate the percentage of BF (%BF) from skinfold thickness requiring measurements at three, four or seven sites, possibly in combination with body circumferences and age^11–16^.

The regression equations proposed by Jackson and Pollock (JP) are based on the assumption that fat is distributed subcutaneously and internally in a similar manner in all individuals^17^. However, it has been shown that fat distribution varies across sex, ethnicity, body type, and age^18–20^. Therefore, the predictive capability of these skinfold regression equations across different populations is limited. Similarly, the regression equations by Durnin and Womersley (DW), Visser (V), Kwok (K) and Gause-Nilsson (GN)^11,14–16^ have been used to predict %BF primarily in healthy older adults, so again caution should be used when extending use in patients.

It is clear that equations may work well on the samples from which they were derived, but there are many aspects of study cohorts that may influence the accuracy of the equation, such as age, obesity, sex, race, ethnicity, and clinical conditions. Several studies have found mixed results in terms of accuracy for several skinfold thickness equations when compared to DXA in different populations^21–28^. Skinfold thickness measurement proved to be an accurate method for determining %BF through the DW equation in overweight patients with chronic obstructive pulmonary disease^29^ and Chambers *et al*.^30^, when studying a sample of older overweight and obese Caucasian American females and males, showed good similarity between DXA and both DW and JP equations. The performance of GN, V and K regression equations in prediction of %BF was inadequate in this case.

These variations should be considered when selecting a skinfold thickness regression equation to estimate %BF. In the absence of studies demonstrating the validity of an equation within a specific population, results are not reliable.

Body composition has been evaluated in a number of studies in patients with diabetes, using different methods^5,31–33^. However, to the best of our knowledge only one study has compared DXA with the skinfold thickness method in the measurement of %BF in these patients^31^. Ingberg *et al*.^31^, showed that %BF was often overestimated through skinfold thickness measurements when compared with estimations by DXA in adolescent girls with type-1 diabetes. Until now, no studies have compared the %BF calculated through skinfold thickness regression equations with %BF measured by DXA in a sample of overweight/obese male and female adult patients with type-2 diabetes. Thus, to date, it is unclear which estimations by skinfold thickness equations may best replicate %BF measured by DXA in typical overweight and obese adult patients with diabetes.

The aim of the present study was to assess whether the equations most commonly used for predicting %BF skinfold thickness may fit with direct measures of %BF obtained by DXA in overweight/obese patients with type-2 diabetes. Further, two predictive equation were developed using DXA measurements as the reference for estimation of percentage total body and trunk fat.

## Results

One participant in the study dropped out early and did not complete all evaluations. Accordingly, data from 39 participants, 12 females and 27 males, were included in the analysis with complete data for all body composition and skinfold thickness variables. Duration of diabetes, metabolic control and lipid profile were comparable in male and female participants (duration of diabetes 10.3 ± 7.5 vs 9.9 ± 5.7 years; HbA1c 7.3 ± 0.7 vs 7.2 ± 0.7%; fasting glucose 164 ± 31 vs 145 ± 27 mg/dL; total cholesterol 170 ± 30 vs 171 ± 33 mg/dL; LDL-cholesterol 94.3 ± 29.6 vs 91.0 ± 18.7; and triglycerides 152 ± 96 vs 126 ± 85 mg/dL, respectively; p = ns for all), whereas HDL-cholesterol was lower in males than in females (46.7 ± 11 vs 54.5 ± 9.4 mg/dL, p < 0.05). The demographic, anthropometric and body composition characteristics of participants are reported in Table 1. No sex differences were found in age, weight and BMI, while height was higher in males than in females.

**Table 1:** Characteristics of the participants enrolled in the study by sex. Data are presented as mean (standard deviation).

**Variable**	**Female (n = 12)**	**Male (n = 27)**
Age, years	56.2 (8.1)	56.6 (7.1)
Weight, kg	77.2 (14.9)	86.2 (13.3)
Height, cm	158.1 (4.6)***	172.9 (4.8)
BMI, kg/m^2^	30.9 (5.4)	28.9 (4.3)
Body fat (DXA), %	37.95 (4.94)***	27.26 (4.95)
Triceps skinfold, mm	22.3 (7.2)***	11.3 (3.5)
Subscapular skinfold, mm	30.3 (11.0)*	20.6 (6.9)
Biceps skinfold, mm	15.6 (5.6)**	8.3 (2.5)
Supra-iliac skinfold, mm	26.7 (8.4)*	20.1 (8.9)

BMI = body mass index; DXA = dual-energy X-ray absorptiometry;

*P < 0.05, **P < 0.01; ***P < 0.001.

In the female group %BF-DXA and skinfold thickness at the triceps, biceps, subscapular, and suprailiac sites were significantly greater in females than in males (Table 1).

A summary of the results related to accuracy and bias in estimates of %BF, with anthropometric equations relative to DXA, is presented in Table 2. In both females and males, the estimate of %BF calculated from DW_B_ was similar to %BF-DXA (1.08%, P = 0.79 and 0.17%, P = 0.23, respectively). However, %BF predicted by DW_S_ was similar to %BF-DXA in males (0.87%, P = 0.17) but not in females (2.75%, P = 0.09). In both females and males, the DW_B_ equation developed the smallest MSD (−0.89% and 0.03%, respectively), while the G equation the highest (10.24% and 6.26%, respectively).

**Table 2:** Summary of the associations between % body fat (%BF) estimated with different anthropometric equations (see text for abbreviations) and the criterion value (obtained using dual-energy x-ray absorptiometry, DXA).

**Females (n = 12)**	Mean ± SD	MSD	R^2^	SEE	E	**Males (n = 27)**	MSD	R^2^	SEE	E
						Mean ± SD				
%BF-DXA	37.95 ± 4.94					27.26 ± 4.95				
%BF-DW_S_	40.70 ± 4.51**	−0.89	0.639	3.11	3.99	28.13 ± 4.42	0.03	0.598	3.20	3.25
%BF-DW_B_	39.03 ± 4.16	−2.76	0.639	3.11	3.05	27.43 ± 4.08	−0.87	0.598	3.20	3.21
%BF-V_S_	45.41 ± 2.66***	−5.23	0.589	3.32	8.13	31.86 ± 2.20***	−3.41	0.601	3.19	5.75
%BF-V_B_	43.38 ± 2.46***	−7.46	0.589	3.32	6.35	30.87 ± 2.03***	−4.60	0.601	3.19	5.05
%BF-G	27.71 ± 5.89***	10.24	0.691	2.88	10.71	21.00 ± 5.36***	6.26	0.708	2.72	6.89
%BF-K	46.61 ± 8.06***	−8.66	0.724	2.72	9.74	33.38 ± 6.40***	−6.12	0.754	2.50	6.90

MSD = mean signed difference; R^2^ = R Square; SEE = standard error of estimate; E = total error; *P < 0.05, **P < 0.01; ***P < 0.001.

The V_S_, V_B_, and K predictive equations significantly overestimated %BF, as compared to DXA, by 7.46%, 5.43% and 8.66% respectively, in females, and by 4.60%, 3.61% and 6.12% respectively, in males (P < 0.001 for all). Conversely, the G equation significantly underestimated %BF compared to DXA in both females and males, by 10.24% and 6.26% respectively, (P < 0.001 for both). As shown in Table 3, SEE ranged from 2.72% to 3.32% in females, and from 2.50 to 3.20 in males. In both sexes, E was lower for DW_B_ compared with the other predictive equations. Overall, %BF estimates showed smaller E in males than females with most predictive equations.

**Table 3:** Equations used to predict percent of body fat (%BF) in overweight/obese patients with type-2 diabetes.

**Reference**	**Equations**
Durnin and Womersley^11^	BD for F = 1.1339 - 0.0645 log10 (Sum 4)
	BD for M = 1.1715 - 0.0779 log10 (Sum 4)
	%BF-DW_S_ = (4.95/BD - 4.50) × 100 (Siri’s equation 37)
	%BF-DW_B_ = (4.57/BD - 4.14) × 100 (Brozek’s equation 38)
Visser *et al*.^14^	BD = 1.0688 + 0.0212 (Sex A) - 0.0356 (log10 [Sum 4])
	%BF-V_S_ = (4.95/BD - 4.50) × 100 (Siri’s equation 37)
	%BF-V_B_ = (4.57/BD - 4.14) × 100 (Brozek’s equation 38)
Gause-Nilsson and Dey^16^	%BF-GN = 36.078 + 3.917 log10(Sum 4) - 5.114(Sex A) + 0.384(W) - 0.289(H)
Kwok *et al*.^15^	%BF-K = −27.149 + 6.137(Sex B) + 1.120 (BMI) + 17.308 log10 (Sum 2)

BD, body density; Sum 4, triceps + biceps + subscapular + suprailliac; Sex A, female = 0, male = 1; Sex B, female = 2, male = 1; Sum 2, triceps + biceps; W, weight (kg); H, Height (cm).

**Figure 1:**
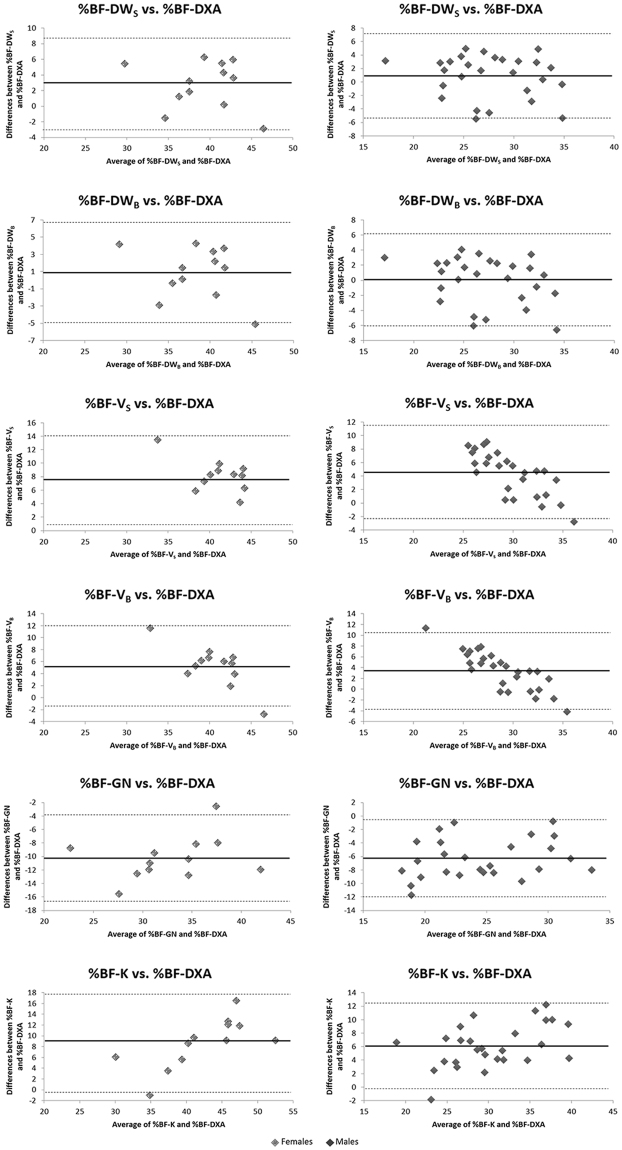
Bland-Altman Plots. %BF, body fat percentage; DXA, dual-energy X-ray absorptiometry; DW_B_, Durnin-Womersley equation for body density and Brozek’s equation to convert body density to %BF; DW_S_, Durnin-Womersley equation for body density and Siri’s equation to convert body density to %BF; V_B_, Visser equation for body density and Brozek’s equation to convert body density to %BF; V_S_, Visser equation for body density and Siri’s equation to convert body density to %BF; G, Gause-Nilsson equation; K, Kwok equation. Solid lines indicate main error, dashed lines indicate ± 2 standard deviations.

Figure 1 shows the agreement of anthropometric equations estimated and DXA-measured %BF by Bland-Altman plots. This analysis confirmed the correspondence between the %BF-DXA and DW_B_ and DW_S_ equations in males, and between the %BF-DXA and DW_B_ in females (Fig. 1). In both groups, at least 91.7% of the data points felt within the 95% limits of agreement in each plots.

In the total sample, entering sex, BMI and the sum of four skinfolds (i.e., triceps, biceps, subscapular and suprailiac skinfolds) in stepwise multiple regression analysis, a statistically significant model was yielded for both total body and trunk %BF (F = 72.73, P < 0.001; F = 47.95, P < 0.001, respectively). The model was:

Total body %BF = 3.071 + 0.211 (triceps skinfold) + 0.756 (BMI) + 6.861 (sex); female = 1; male = 0; adjusted R^2^ = 0.850, SEE = 2.71.

Trunk %BF = 9.334 + 0.168 (suprailiac skinfold) + 0.629 (BMI) + 6.536 (sex); female = 1; male = 0; adjusted R^2^ = 0.788, SEE = 3.01.

For the total body %BF and the trunk %BF equation, the Durbin-Watson was 2.23 and 2.17, respectively, indicating that there was no autocorrelation between the residuals and the variance inflation factor was <3.1 and <2.8, respectively, for all variables showing that each model was robust to collinearity.

## Discussion

In this study, we assessed the reliability of %BF estimates obtained with several anthropometric equations using DXA-measured %BF as the criterion in overweight and obese adult patients with type-2 diabetes.

We used DXA as the reference technique to provide an accurate measure of body fat percentage. DXA measurements are greatly reproducible and the validity of this method has been previously demonstrated in different populations^34–37^.

The main finding of the study was the weakness of skinfold thickness anthropometric predictive equations in estimating %BF as compared to DXA in both sexes. In fact, the DW was the only regression equation that adequately predicted %BF in overweight and obese adult patients with type-2 diabetes.

In particular, the DW_B_ equation in females, and both DW_S_ and DW_B_ equations in males gave results that were not significantly different from those obtained by DXA. These results were confirmed by Bland-Altman analysis (Fig. 1), which showed good agreement between %BF-DXA and %BF-DW_B_ equation in females and with both %BF-DW_B_ and %BF-DW_S_ equations in males. This lack of predictive ability for %BF-DW_S_ equation in females may be due to differences in the pattern of body fat distribution between sexes^38^. In this regard, skinfold thickness at the triceps, biceps, subscapular, and suprailiac sites was greater in females than in males (Table 1). In addition, %BF-DXA was also higher in females, although BMI was similar between sexes. The other anthropometric equations investigated in this study, i.e. V_S_, V_B_, and K equations, significantly overestimated, whereas G underestimated %BF. Interestingly, these differences between DXA and the predictive anthropometric equations used in our study we have found in type-2 diabetes patients were also reported in a study carried out in nondiabetic overweight and obese subjects^30^.

Recently, Chambers *et al*.^30^ showed that equations from DW and V accurately predicted %BF in obese females but overestimated %BF in overweight and obese males. Moreover, in this sample, the K equation overestimated fat mass in both males and females, and the G equation underestimated %BF in females, but overestimated it in males. Unfortunately, the study from Chambers *et al*.^30^ did not report the performance of the DW and V equations using the Brozek equation, thereby preventing full comparison with our findings.

Some authors reported a low accuracy of %BF predicted by DW equation in obese people^21,22^, using either bioelectrical impedance or underwater weighing as a reference. Reasons underlying these differences with our findings are not easily explained. In these latter studies, only obese and not overweight individuals were studied.

It appears obvious that obesity may affect the accuracy of skinfold thickness assessment, possibly due to differences in body fat distribution. Notably, some methodological aspects may contribute to limiting the use of skinfold thickness measurements in obese subjects. For example, the calipers currently used are not large enough to pinch and measure certain body sites such as the abdominal, suprailiac or thigh areas in obese subjects. Another important factor is that raising skinfolds for measurement is more difficult in obese people. Proper technique requires pinching a fold of skin and fat between the thumb and forefinger and separating them from the underlying muscle^21^, this process being subject to considerable variation between investigators. Thus, either over or underestimation by skinfold thickness measurement is possibly due not only to the characteristics of subjects investigated, but also to the technical ability of data collectors.

In order to offer a practical tool for %BF estimation in type-2 diabetic patients, a predictive equation was developed regressing DXA-measured %BF against easy-to-measure variables such as sex, BMI, and skinfold thicknesses. The resulting model included BMI, triceps skinfold, and sex, all showing positive contribution to %BF. The percentage of explained variance in the dependent variable was high (85%) and the SEE was small (2.71%) suggesting that the equation has good accuracy in predicting %BF in type-2 diabetic patients. Given the relevance of visceral fat as a health risk factor in overweight, obese and type-2 diabetes people, a predictive equation was also developed using DXA-measured trunk %BF, a proxy of visceral fat^39^ as the dependent variable and the same above anthropometric measurement as independent variables. The resulting model included BMI, suprailiac skinfold, and sex, all showing positive contribution to trunk %BF. The percentage of explained variance (78.8%) and the SEE (3.01) were such that the equation could be considered fairly good accuracy in predicting the percentage of centrally located fat. However, both models should be used with some caution in practice because of the small number of patients and the lack of a cross-validation sample.

Our study has some limitations that should be acknowledged; first, we did not investigate a non-diabetic control group. This aspect precluded us from assessing whether there may be specific differences between diabetic and non-diabetic subjects. Patients with type-2 diabetes are typically characterized by central fat distribution, with the presence of ectopic fat depots. Therefore, skinfold thickness might predict fat mass to different extents in people with or without diabetes. While this issue should be further explored in future studies, our data are important in giving information about the more appropriate equations for estimating fat mass in diabetic patients through simple skinfold thickness measurement. Another limitation is related to the homogeneous characteristics of the diabetic subjects recruited in this study, who were all older Caucasian adults, mostly overweight or obese. Thus, some caution should be taken when extrapolating data from the present study to diabetic patients with different characteristics. Nevertheless, our sample made up of overweight and obese adult patients does reflect the clinical features of most typical patients with type-2 diabetes.

Conversely, the main strengths of our study are the use of DXA as the reference standard, which is a well-accepted method providing an accurate assessment of fat mass, and the measurement of skinfold thickness by a trained operator.

In conclusion, estimating fat mass is important in the management of type-2 diabetes. While most of the equations derived from skinfold thickness over or underestimated %BF in a cohort of overweight/obese diabetic patients, DW equation gave reliable results. Further, a new well-performing predictive equation for %BF was developed in our sample of overweight and obese adult patients with type-2 diabetes using the accurate DXA measurements as the dependent variable. Hopefully, this information will assist clinicians and nutritionists in assessing fat mass in their patients through skinfold thickness equations. However, further research in a larger number of patients is needed in order to confirm the validity of anthropometric equations in this population.

## Methods

According to Chow and colleagues^40^, the required sample size was estimated “a priori” using the formula: $n\,=\,{({z}_{\alpha /2}+{z}_{\beta })}^{2}{\sigma }_{m}^{2}/{\epsilon }^{2}$, where $\epsilon$ is the mean value of the differences between the %FM calculated with DXA and the %FM calculated from skinfolds equations and ${\sigma }_{m}$ was the standard deviation of these differences. Preliminary investigation showed that ${\sigma }_{m}$ was approximately 4% in each experimental group. Setting the minimum clinically meaningful difference at 2% (effect size = $\,|{\epsilon }|/{\sigma }_{m}\approx 0.5$) and the type I error at α = 0.05, the minimum sample size required for having an 80% power (i.e., β = 0.20) was 34 subjects.

Accordingly, the study was carried out on baseline data from a total of 40 (12 females and 28 males) sedentary patients with type-2 diabetes, aged 40–68 years, recruited to participate in the RAED2 Study, a randomized controlled trial aimed at comparing the metabolic effects of aerobic and resistance training in diabetic subjects^41^). Inclusion criteria were type-2 diabetes for at least 1 year, hemoglobin (Hb)A1c 6.5–9.0% (reference limit, <6.5%), baseline physical activity <1,000 MET min per week by the International Physical Activity Questionnaire (IPAQ). Allowed diabetes medications were oral hypoglycemic agents. Exclusion criteria were moderate/severe somatic or autonomic neuropathy, cardiovascular disease, preproliferative/proliferative retinopathy, chronic renal failure, as well as therapy with beta-blockers or current smoking. All female participants were postmenopausal and not on hormonal replacement therapy. All methods were carried out in accordance with relevant guidelines and regulations. This study was approved by the Ethical Committee of the Azienda Ospedaliera Universitaria Integrata Verona, Verona, Italy, which was according to the Helsinki Declaration (revised 2008). Written informed consent was obtained from all the participants.

### Anthropometry and body composition analysis

Body mass was taken at the nearest 0.1 kg with an electronic scale (Tanita electronic scale BWB-800 MA); stature was measured with a Harpenden stadiometer (Holtain Ltd., Crymych, Pembs. UK) to the nearest 0.5 cm; body mass index (BMI) was calculated as weight (kg)/height (m)^2^. Whole body and regional body composition was evaluated using a total body scanner (QDR Explorer W, Hologic, MA, USA; fan-bean technology, software for Windows XP version 12.6.1), according to the manufacturer’s procedures. Instrument quality control (QC) of the DXA scanner was performed daily before actual use by means of the Hologic encapsulated spine phantom (Hologic Inc, Bedford, MA) provided by the manufacturer. Over the study period, the Hologic software always passed the daily QC calibration and the phantom measurements (i.e., bone mineral density, bone mineral content and area values) always fell within the control limit of upper and lower ranges set as a baseline ± 1.5%. Furthermore, the long-term DXA scanner performance was monitored over the entire study period during which the measurements from phantom scans remained stable without drifting and with a coefficient of variation lower than 1% for each of the phantom measurements. All scans were performed and analyzed by the same operator (CM), in order to ensure consistency. The DXA-derived percentage of body fat (%BF) at the total body level (%BF-DXA) as well as in the trunk region were included in the analysis. The Hologic software includes the neck, chest, abdominal and pelvic areas in what is defined as the trunk region. Its upper perimeter is the inferior edge of the chin and the lower boundary intersects the middle of the femoral necks without touching the brim of the pelvis. The trunk region is separated from the arm region at the glenohumeral joint.

A trained operator (CM) performed skinfold thickness measurements twice with a Harpenden caliper (Gima, Milan, Italy) on the subject’s right side at the triceps, biceps, subscapular, and suprailiac sites, according to standard procedures^42,43^. If the two measurements differed by more than 2 mm, a third measurement was taken, and the two closest were then averaged and recorded to the nearest 0.1 mm as the final value. Established age and sex-specific prediction equations, based on DW^11^ and V^14^, were used to estimate body density (BD), and BD values were then converted to %BF according to both Siri^44^ and Brozek^45^ equations (DWS, DWB, VS, and VB, respectively) (Table 1). In addition, %BF was also estimated using the prediction equations developed by Gause-Nilsson and Dey^16^ (G), and by Kwok *et al*.^15^ (K) (Table 3). Accordingly, %BF estimated from six predictive equations (%BF-DWS, %BF-DWB, %BF-VS, %BF-VB, %BF-G and %BF-K) were compared to %BF-DXA (Table 3). The anthropometric prediction equations to be compared with DXA were selected for the following reasons. Firstly, the selected equations are the most commonly used in the clinical setting to predict %BF in older adults. In particular, the age- and sex-specific DW equation^11^ is widely used in Europe^46^. Secondly, the selected equations use a small number of anthropometric measurements to assess %BF, which is saving clinicians and researchers time and resources. Thirdly, several skinfolds used in other predictive equations (e.g., the abdominal skinfold) are difficult to measure in overweight and obese subjects because of the inadequate size of most calipers (upper measurement limit of 45 to 55 mm) along with the difficulty of grasping and holding a large skinfold while reading the caliper dial.

### Biochemical analyses

Hemoglobin A1c was measured by a Diabetes Control Complications Trial (DCCT)-aligned method, with an automated high-performance liquid chromatography analyzer (Bio-Rad Diamat, Milan, Italy). Serum total cholesterol, HDL-cholesterol and triglycerides were determined by standard laboratory procedures (DAX 96; Bayer Diagnostics, Milan, Italy). LDL-cholesterol was calculated using Friedewald’s equation^47^.

### Statistical analysis

Descriptive statistics (mean and standard deviation) were computed by sex for all variables using standard procedures. Normality of data was assessed with the Kolmogorov-Smirnov test. Sex comparisons were made using independent-sample t tests. Paired-sample t tests were performed to assess whether %BF skinfold thickness values were accurate in comparison with DXA. The mean signed difference (MSD i.e., the average of the differences between the skinfold thickness estimated %BF and the criterion [DXA] measurement) was also computed to test the ability of anthropometric equations to accurately estimate the mean level of %BF^48^.

The coefficient of determination (R^2^) and the standard error of estimate (SEE) for %BF-DXA and equation-derived %FM values were computed to test agreement between methods. The total error (total error $[{\rm{E}}]=\sqrt{\sum ({\rm{Y}}^{\prime} -{\rm{Y}}}{)}^{{\rm{2}}}/{\rm{n}}$, where Y′ = the predicted %BF values from the skinfold regression equation, Y = the criterion %BF values from DXA, and n is the number of participants) was calculated according to Lohman^49^. The visual absolute agreement (bias and limits of agreement) between the %BF resulting from predicting equations (i.e. %BF-DWS, %BF-DWB, %BF-V_S_, %BF-V_B_, %BF-G and %BF-K) and %BF-DXA was evaluated assessing the Bland and Altman plots^50^.

A stepwise multiple regression analysis on the total sample with %BF-DXA as dependent variable and sex, BMI and the four skinfolds (i.e., triceps, biceps, subscapular and suprailiac skinfolds) as potential predictors was carried out. Adjusted R^2^ and SEE were used to represent the goodness of the predictor model. Homoscedasticity of data was assessed by plotting the residuals of multiple regression analysis against the predicted values. The presence of serial correlations among the residuals was tested using the Durbin-Watson statistic and the variance inflation factor was calculated to check for multicollinearity in our multiple linear regression model.

Statistical analyses were performed using SPSS v. 16.0 (IBM Corp., Armonk, NewYork, USA), alpha value being set at 0.05.

### Data availability

The dataset generated and analyzed during the current study is not publicly available because it refers to personal and delicate data of our patients. It is available from the corresponding author on reasonable request.
